# Oxaliplatin Induces Immunogenic Cell Death in Human and Murine Laryngeal Cancer

**DOI:** 10.1155/2022/3760766

**Published:** 2022-09-12

**Authors:** Jingmiao Wang, Haizhong Zhang, Xiaoyan Yin, Yanrui Bian

**Affiliations:** The First Department of Otorhinolaryngology, The Second Hospital of Hebei Medical University, No. 215 Heping West Road, Shijiazhuang 050000, Hebei, China

## Abstract

**Background:**

Cisplatin resistance is observed in patients with laryngeal cancer. The present study was designed to explore the efficacy of oxaliplatin on laryngeal cancer and elucidate the underlying mechanisms.

**Methods:**

Cell viability was determined by using MTT assays. Cell apoptosis was determined by using annexin V and propidium iodide (PI) staining. Flow cytometry and immunofluorescence were applied to determine the levels of calreticulin (CALR) and DiD (1,1-dioctadecyl-3,3,3,3-tetramethylindodicarbocyanine). Flow cytometry was applied to analyze the levels of CD83, CD86, IFN-*γ*-producing CD8^+^ T cells, and CD4^+^CD25^+^FoxP3^+^ Tregs. The levels of adenosine triphosphate (ATP) were determined by using a chemiluminescent ATP kit and cytokines were determined by using specific enzyme-linked immunosorbent assays (ELISAs). The levels of HMGB1 were determined by using Western blot and ELISA, respectively. The xenograft animal model was constructed to evaluate the antitumor effects of oxaliplatin.

**Results:**

Oxaliplatin inhibited cell growth, promoted cell apoptosis, and induced the levels of CALR, ATP, and high mobility group box protein 1 (HMGB1) in Hep-2 cells. Oxaliplatin-treated Hep-2 cells increased the intensity of DiD and the levels of CD83 and CD86 in dendritic cells (DCs), as well as induced the supernatant IL-6 and TNF-*α*. Oxaliplatin-treated primary laryngeal cancer cell-pulsed DCs increased the IFN-*γ*-producing CD8^+^ T cells and suppressed CD4^+^CD25^+^FoxP3^+^ Tregs. *In vivo* data showed that oxaliplatin suppressed tumor growth and increased the populations of CD86^+^CD80^+^ and CD8^+^CD45^+^ cells in the tumor tissues.

**Conclusion:**

Treatment with oxaliplatin inhibited laryngeal cancer cells by inducing immunogenic cell death.

## 1. Introduction

Laryngeal cancer is a malignancy in the upper digestive and respiratory tract [1]. According to the Global Cancer Statistics in 2018, 177,422 new cases were diagnosed with laryngeal cancer, and 94,771 deaths were caused by laryngeal cancer, accounting for 1% of newly diagnosed cancer cases and related deaths [2]. Laryngeal cancer can be attributed to various factors including inherited gene defects (mutation), tobacco and alcohol use, human papillomavirus infection, and nutritional deficiencies [3]. The laryngeal cancer stage ranges from I to IV. Besides, the vast majority (> 97%) of laryngeal cancers are squamous cell carcinoma [1].

Currently, the treatment options are based on the UICC TNM 8th edition staging system [4, 5]. The standard therapy for laryngeal cancer in the early stage (stage I or II) includes conservative surgery or radiation therapy [4, 6]. When laryngeal cancer is in the advanced stage (Stage III or IV), therapy options are surgery plus chemoradiotherapy or CRT alone [5]. In brief, therapeutic strategies for patients with advanced laryngeal cancer include chemotherapy, radiotherapy, surgery, or a combination of these [7, 8]. However, it is difficult to treat laryngeal cancer at the advanced stage [9].

Platinum-based chemotherapy is used as the first-line treatment [10]. However, platinum resistance is one of the major challenges in laryngeal cancer therapy [11]. For instance, cisplatin is used in patients with advanced laryngeal cancer or who received surgery at recurrence and achieved satisfactory effects in the initial stage [12]. However, patients who initially respond to cisplatin therapy often develop acquired drug resistance after long-term exposure to cisplatin.

Unlike cisplatin, oxaliplatin exhibits the same potency but with a better safety profile and less platinum resistance [13]. Chemotherapeutic mechanisms of platinum are in part achieved by inhibiting DNA synthesis and inducing cell apoptosis [14]. Unlike cisplatin, oxaliplatin exhibits distinct pharmacological and immunological properties, which feature the bidentate ligand 1,2-diaminocyclohexane in place of two monodentate ammine ligands [15]. Interestingly, previous studies have revealed that oxaliplatin not only regulates cancer cell apoptosis but also induces immunogenic cell death (ICD) [16]. ICD triggers immune responses in the tumor microenvironment through a series of events [17, 18]. First, the occurrence of ICD is accompanied by the release of adenosine triphosphate (ATP), which serves as the “find me” signal for dendritic cells (DCs) [18]. Second, ICD leads to the translocation of calreticulin (CRT) into the surface of cancer cells, which releases the “eat me” signal for DCs [18]. Third, a large amount of high mobility group box 1 (HMGB-1) released during ICD binds to the toll-like receptors on the immune cells (DCs and macrophages), which also benefits the antigen-presenting functions of DCs [18]. Consequently, tumor-specific T cells are induced and T cell-mediated immune responses are triggered [18, 19]. To our knowledge, the effects of oxaliplatin on laryngeal cancer and its underlying mechanisms are still unclear. Therefore, this study, for the first time, investigated the antitumor effects of oxaliplatin in laryngeal cancer. Moreover, we elucidated whether the underlying mechanisms of Oxaliplatin on laryngeal cancer were related to ICD.

## 2. Methods and Materials

### 2.1. Reagents and Antibodies

Cisplatin (purity 99%) and oxaliplatin (purity 99%) were purchased from Boyuan Chemical Company (Nantong, China) and dissolved in sterile water. MTT (3-[4,5-dimethylthiazol-2-yl]-2,5 diphenyl tetrazolium bromide) assay kit and crystal violet were purchased from Sangon Biotech (Shanghai, China). ATP determination kit was purchased from Thermo Fisher (MA, United States). A cell apoptosis kit (PI-annexin V) was purchased from BD (NJ, United States). APC-conjugated calreticulin (CALR) antibody was purchased from Biorbyt (MO, United States). HMGB-1 antibody was purchased from Abcam (MA, United States). FITC-conjugated anti-CD83 (Clone: Michel-19), BV510 conjugated anti-CD86 (Clone: IT2.2), PerCP-Cy5.5 conjugated anti-CD8 (Clone: RPA-T8), BV421 conjugated anti-CD4 (Clone: OKT4), AF700 conjugated anti-IFN-*γ* (Clone: 4S.B3), and PE-conjugated anti-FoxP3 (Clone: 206D) were purchased from Biolegend (CA, United States). Vybrant™ DiD Cell-Labeling Solution was purchased from Invitrogen (CA, United States).

### 2.2. Cell Culture and Cell Viability Assays

Human laryngeal cancer cells Hep-2 and mouse head and neck carcinoma cells SCC7 were purchased from ATCC and cultured in Eagle's Minimum Essential Medium supplement with 10% fetal bovine serum (FBS). Hep-2 cells were seeded at a density of 5,000 cells per well in a 96-well microplate and incubated for 24 h. Next, cisplatin (5, 7.5, and 10 *μ*M) or oxaliplatin (5, 7.5, and 10 *μ*M) was added and incubated for another 48 h. Cell viability was determined by the MTT assay.

### 2.3. Quantification of ATP and HMGB1

After 24 h exposure to different treatments, cell supernatant was collected and centrifuged at 15000 g for 30 mins to separate the proteins. A Luminometric ATP Assay Kit (AAT Bioquest, CA, USA) was then utilized to quantify the content of ATP in the supernatant. Mixed ATP monitoring enzyme, ATP sensor, and reaction buffer were used to prepare the ATP assay solution in accordance with the manufacturer's instructions. Following exposure to different treatments, the samples were mixed with the ATP assay solution to detect the intensity of luminescence. The concentrations of ATP were then calculated. Western blot was used to detect the release of HMGB1 in cell supernatant. BSA (bovine serum albumin) in the culture medium was used as a loading control.

### 2.4. Cell Apoptosis

Hep-2 cells were seeded at a density of 1.0 × 10^5^ cells per well in 24-well plates. Next, the cells were treated with cisplatin (5, 7.5, and 10 *μ*M) or oxaliplatin (5, 7.5, and 10 *μ*M) for 24 h. Cell suspension was prepared and incubated with FITC-conjugated annexin V staining solution. Cell suspension was stained with the PI solution in the dark. The population of early apoptotic and late apoptotic cells were analyzed using a flow cytometer (BD Bioscience, CA, United States).

### 2.5. Isolation of Dendritic Cells (DCs)

DCs were isolated from peripheral blood mononuclear cells (PBMCs) according to the previously described method [20]. In brief, the PBMCs were isolated enriched by gradient centrifugation, and monocytes were depleted using RosetteSep™ Human Monocyte Depletion Cocktail (STEMCELL, USA).

### 2.6. Flow Cytometry

Hep-2 cells were seeded at a density of 1.0 × 10^5^ cells per well in 12-well plates. After treatment with cisplatin (7.5 *μ*M) or Oxaliplatin (7.5 *μ*M) for 24 h, the cells were collected. Cells were stained with APC-conjugated anti-CALR and PI and then analyzed by a flow cytometer.

Hep-2 cells were treated with cisplatin (7.5 *μ*M) or oxaliplatin (7.5 *μ*M) for 24 h prior to coculturing with DCs that were isolated from PBMCs. The ratio of DCs and Hep-2 cells was 5 : 1. After coculturing for 4 days, total cells were collected, and DCs were stained with FITC-conjugated anti-CD83 and BV510-conjugated anti-CD86. The population of DCs and mean fluorescence intensity were analyzed by flow cytometer (BD LSRFortessa, BD Biosciences, USA).

## 3. Evaluation of DCs Phagocytosis

Hep-2 cells were treated with cisplatin (7.5 *μ*M) or oxaliplatin (7.5 *μ*M) for 24 h prior to coculturing with DCs that were isolated from PBMCs. The ratio of DCs and Hep-2 cells was 5 : 1. After coculturing for 4 days, DC phagocytosis was evaluated. DCs were collected and stained with DiD labeling solution and analyzed by a flow cytometer. In addition, DCs were stained with DiD labeling solution.

### 3.1. ELISA

Hep-2 cells were treated with cisplatin (7.5 *μ*M) or oxaliplatin (7.5 *μ*M) for 24 h prior to coculturing with DCs that were isolated from PBMCs. The ratio of DCs and Hep-2 cells was 5 : 1. After coculturing for 4 days, cell supernatant was collected. The levels of interleukin (IL)-6 and tumor necrosis factor (TNF)-*α* were determined using specific ELISA kits (R&D Biosystem, MN, United States).

### 3.2. Evaluation of Tumor-Specific T Cells

Primary laryngeal cancer cells were treated with cisplatin (7.5 *μ*M) or oxaliplatin (7.5 *μ*M) for 24 h prior to coculturing with DCs that were isolated from PBMCs. The ratio of DCs and Hep-2 cells was 5: 1. DCs were collected after coculturing for 4 days and referred to as cocultured DCs. Unpulsed DCs (DCs without coculturing) were used as the control group. Cocultured or unpulsed DCs were added into autologous T cells and cultured for 14 days. After that, T cells were collected and stained with PerCP-Cy5.5-conjugated anti-CD8, BV421-conjugated anti-CD4, AF700-conjugated anti-IFN-*γ*, and PE-conjugated anti-FoxP3. A flow cytometer was applied to analyze the population of IFN-*γ*-producing CD8^+^ T cells and CD4^+^FoxP3^+^ Tregs cells. CD8^+^ T and CD4^+^ T cells were gated from the cocultured cells according to the staining of the anti-CD8 antibody and anti-CD4 antibody.

### 3.3. Xenograft Animal Model Construction and Drug Administration

Nude mice were purchased from Shanghai Model Organisms (Shanghai, China). Studies were approved by the Second Hospital of Hebei Medical University. After adaption for one week, the mice were subcutaneously injected with 2 × 10^6^ SSC7 cells. When tumor volumes reached about 50 mm^3^, the mice were divided into three groups. In the cisplatin-treated group, the mice received cisplatin (3.0 mg/kg, i.v.) every three days for 4 times. In the oxaliplatin-treated group, the mice received oxaliplatin (3.0 mg/kg, i.v.) every three days for 4 times. In the control group, the mice received an equal volume of PBS every three days for 4 times. Tumor growth was evaluated in the experimental period. At the end of the experimental period, the mice were euthanized and the tumor tissues were collected. The cell suspension was prepared to analyze the populations of CD86^+^CD80^+^ and CD8^+^CD45^+^ cells in tumor tissues.

### 3.4. Statistical Analysis

Data were shown as mean ± standard deviation (S.D). Statistical analysis was performed using GraphPad Prism8 (GraphPad Software; La Jolla, CA, USA) using a Student's *t*-test or one-way analysis of variance with multiple comparisons. A *p* value less than 0.05 was considered statistically significant.

## 4. Results

### 4.1. Inhibitory Effects of Cisplatin and Oxaliplatin on Human Hep-2 Laryngeal Cancer Cell Growth

**Figure 1:**
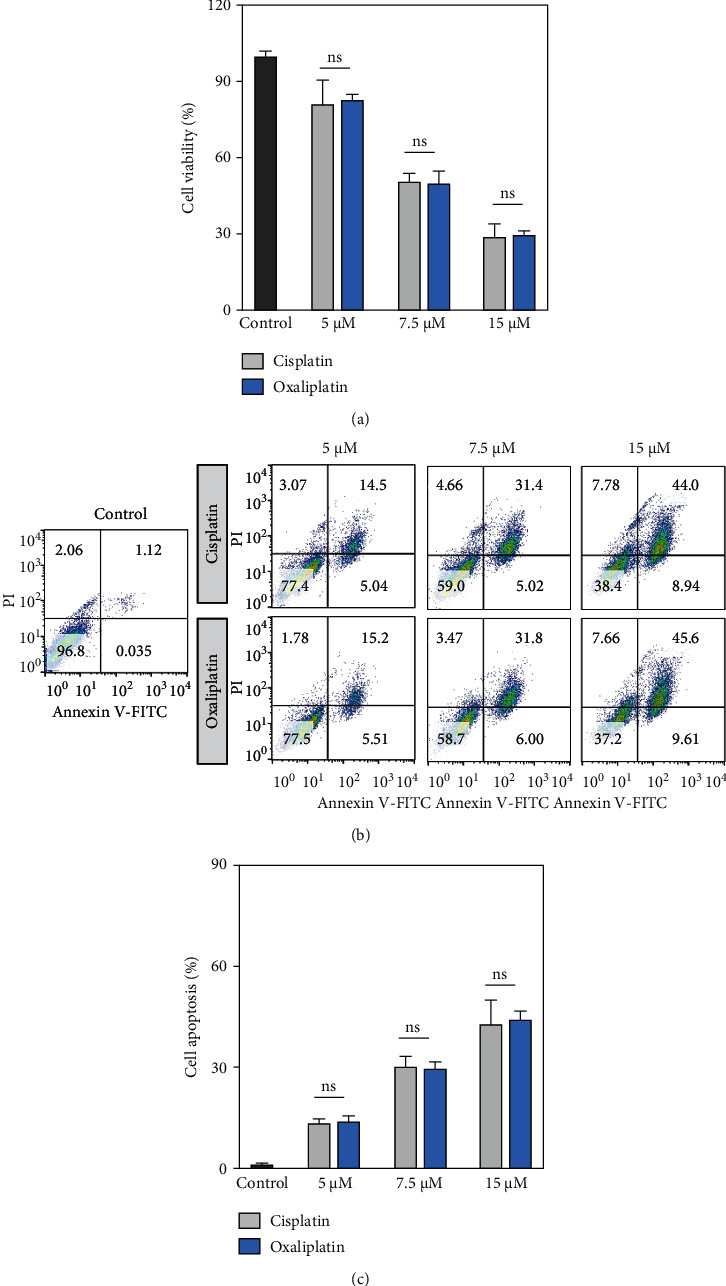
Inhibitory effects of cisplatin and oxaliplatin on human Hep-2 laryngeal cancer cell growth. (a) The cells were incubated with cisplatin or oxaliplatin (5, 7.5, and 10 *μ*M) for 48 h Cell viabilities were determined by MTT assay. (b) Cell apoptosis was determined by propidium iodide and annexin V staining. (c) The population of apoptotic cells including early- and late-apoptosis (annexin V^+^PI^−^ and annexin V^+^PI^+^, respectively), and/or necrotic cells were analyzed by flow cytometer. Data were represented as the means ± SD. ns indicates no significant difference.

The effects of cisplatin and oxaliplatin on the viability of Hep-2 cells were determined by MTT assay. We found that treatment with cisplatin or oxaliplatin significantly decreased the cell viability in a concentration-dependent manner (Figure 1(a)). Moreover, the effects of cisplatin and oxaliplatin on cell apoptosis were determined by a flow cytometer. We observed that treatment with cisplatin and oxaliplatin significantly increased the populations of apoptotic cells as compared to the control group (Figure 1(b)). However, the percentages of apoptotic cells, and/or necrotic cells, between cisplatin-treated and oxaliplatin-treated groups showed no significant difference (Figure 1(c)). These results supported that cisplatin and oxaliplatin exhibited similar antitumor effects against Hep-2 cells.

## 5. Oxaliplatin-Induced Immunogenic Cell Death (ICD) in Hep-2 Cells

**Figure 2:**
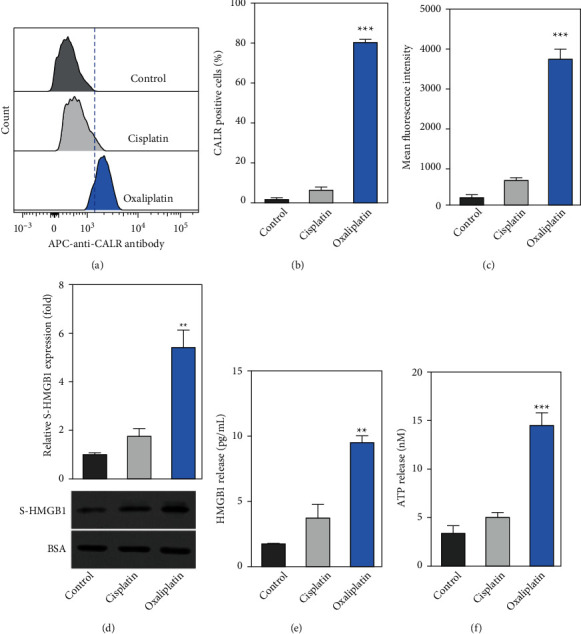
Oxaliplatin induced immunogenic cell death (ICD) in Hep-2 cells. (a) After the cells were incubated with cisplatin (7.5 *μ*M) or oxaliplatin (7.5 *μ*M) for 24 h the cells were then stained with the APC-labeled anti-CALR antibody. The levels of surface calreticulin (CALR) in viable cells (refers to as PI negative) were determined by a flow cytometer. (b-c) The percentage of CALR-positive cells and the mean fluorescence intensity (MFI) were determined by flow cytometry. (d-e) Hep-2 cells were incubated with cisplatin or oxaliplatin. Next, the levels of HMGB1 in cell supernatant were determined by Western blotting and ELISA, respectively. (f) ATP released was determined by a commercialized kit. Data were represented as the means ± SD. ^*∗∗*^*p* < 0.01, ^*∗∗∗*^*p* < 0.001 compared with the cisplatin group.

Next, we evaluated the effects of cisplatin and oxaliplatin on ICD in Hep-2 cells. We found that treatment with oxaliplatin (7.5 *μ*M) significantly increased the population of CALR-positive cells, as well as the MFI (mean fluorescence intensity), as compared to the control group (Figures 2(a)–2(c)). However, cisplatin did not significantly affect the population of CALR-positive cells or MFI as compared to the control group (Figures 2(a)–2(c)). In addition, we also found that oxaliplatin significantly increased the levels of CALR in AMC-HN-8 cells (Fig.  S1).

The levels of supernatant HMGB1 (S-HMGB1) were determined by Western blot and ELISA. We found that treatment with oxaliplatin (7.5 *μ*M) significantly increased the levels of S-HMGB1 as compared to the control group (Figures 2(d) and 2(e)). Consistently, treatment with oxaliplatin (7.5 *μ*M) significantly increased the expression of S-HMGB1 in AMC-HN-8 cells (Fig.  S1B). Furthermore, the amount of ATP released in the supernatant was detected. We observed that treatment with oxaliplatin (7.5 *μ*M) significantly increased the ATP release as compared to the control group (Figure 2(f)). In addition, treatment with oxaliplatin (7.5 *μ*M) also significantly increased the ATP release in AMC-HN-8 cells (Fig.  S1C). These results suggested treatment with oxaliplatin-induced ICD.

## 6. Oxaliplatin-Treated Hep-2 Cells Enhanced DC Phagocytosis and the Levels of Maturation-Associated Markers

**Figure 3:**
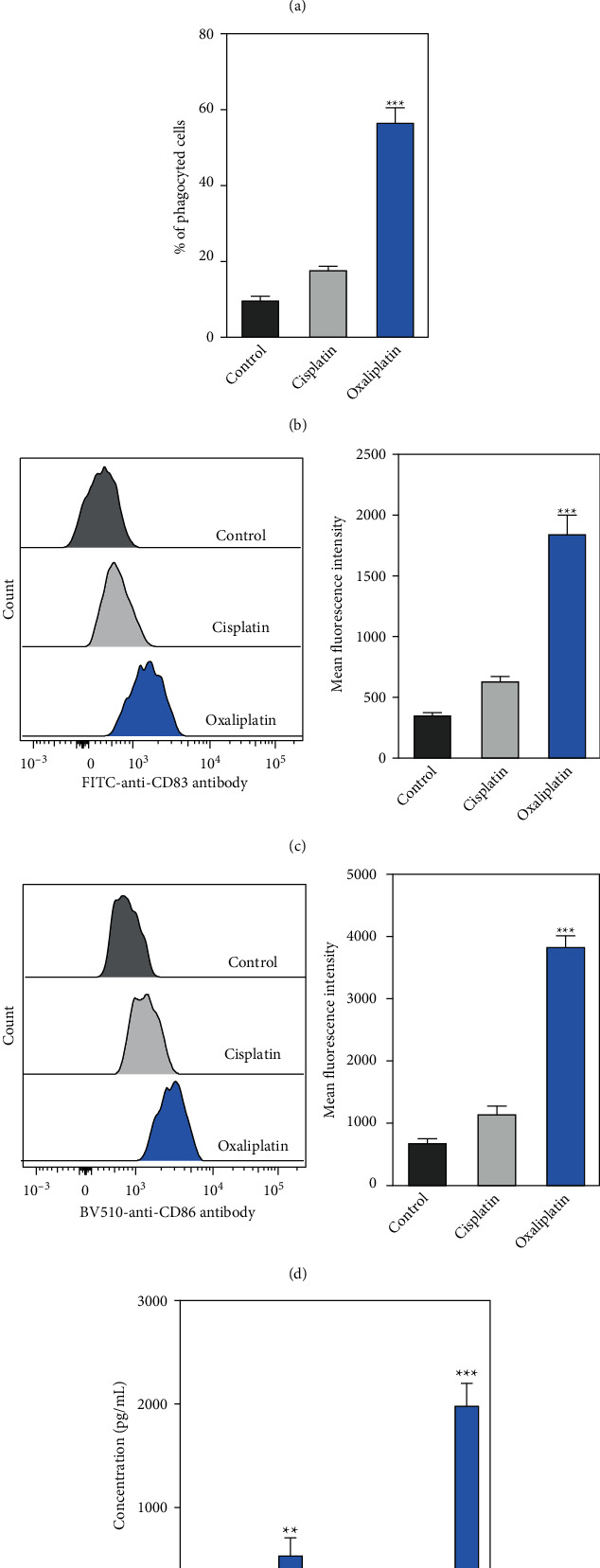
Oxaliplatin-treated Hep-2 cells enhanced DCs phagocytosis and the levels of maturation-associated markers. (a-b) Hep-2 cells were incubated with cisplatin (7.5 *μ*M) or oxaliplatin (7.5 *μ*M) for 24 h Next, the cells were labeled with DiD prior to coculturing with immature DCs. After the cells were co-cultured for 24 h, the phagocytosis of DCs against Hep-2 cells and the percentage of phagocyte cells were determined by immunofluorescence staining. The cells were analysed by flow cytometry. (c-d) The levels of maturation-associated surface markers including CD83 and CD86 were determined by flow cytometry. (e) The levels of IL-6 and TNF-*α* in the supernatant were determined by specific ELISAs. Data were represented as the means ± SD. ^*∗∗*^*p* < 0.01, ^*∗∗∗*^*p* < 0.001 compared with the cisplatin group.

The effects of oxaliplatin-treated Hep-2 cells on DC phagocytosis were determined. We observed that treatment with oxaliplatin significantly enhanced the DiD signal intensity to 83.7% as compared to the control (8.1%) and cisplatin-treated groups (14.3%) (Figure 3(a)). In addition, treatment with oxaliplatin (7.5 *μ*M) also increased the percentage of phagocyted cells as compared to the control and cisplatin-treated groups (Figure 3(b)).

Furthermore, DC maturation-related biomarkers including CD83 and CD86 were determined. Treatment with oxaliplatin (7.5 *μ*M) significantly increased the expression levels of CD83 and CD86 compared to the control and cisplatin-treated groups (Figures 3(c) and 3(d)). We also measured the levels of inflammatory cytokines (IL-6 and TNF-*α*) in the supernatant, which were significantly increased in the supernatant when DCs were cocultured with oxaliplatin-treated Hep-2 cells (Figure 3(e)). Taken together, these results supported that oxaliplatin-treated Hep-2 cells enhanced DC phagocytosis and the levels of DC maturation-associated markers.

### 6.1. The Effects of Oxaliplatin-Treated Primary Laryngeal Cancer Cell Pulsed DCs on the Populations of T Cells

**Figure 4:**
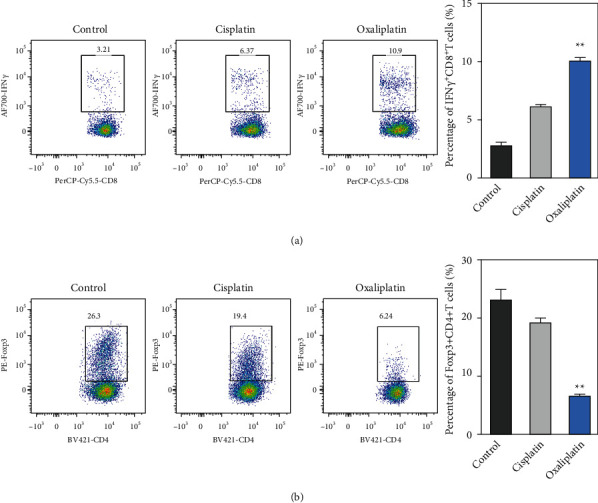
Oxaliplatin-treated primary laryngeal cancer cells pulsed with DCs induced the populations of tumor-specific CD8^+^ T cells and reduced the population of Treg cells. Cisplatin or oxaliplatin-treated primary laryngeal cancer cells were pulsed with monocyte-derived DCs. After that, the cells were used to stimulate autologous T cells for 2 weeks. (a-b) The numbers of IFN-*γ*-producing CD8^+^ T cells were analyzed by intracellular IFN-*γ* staining after stimulation. The frequencies of CD4^+^CD25^+^FoxP3^+^ Treg cells were analyzed by a flow cytometer. The results are from five independent studies. Data were represented as the means ± SD. ^*∗∗*^*p* < 0.01 compared with the cisplatin group.

Finally, we evaluated the effects of DCs that were cocultured with oxaliplatin-treated primary laryngeal cancer cells on the populations of T cells, including IFN-*γ*-producing CD8^+^ T cells and CD4^+^CD25^+^FoxP3^+^ Treg cells. Primary laryngeal cancer cells were treated with cisplatin (7.5 *μ*M) or oxaliplatin (7.5 *μ*M) prior to coculturing with DCs. Cocultured or unpulsed DCs were added into autologous T cells and incubated for 14 days. We observed that cocultured DCs significantly increased the populations of IFN-*γ*-producing CD8^+^ T cells (Figure 4(a)) and suppressed the populations of CD4^+^CD25^+^FoxP3^+^ Treg cells (Figure 4(b)). These results demonstrated that the antitumor effects of oxaliplatin were in part mediated by the regulation of tumor-specific T cells.

### 6.2. Treatment with Oxaliplatin Suppressed Tumor Growth in Xenograft Animal Model

**Figure 5:**
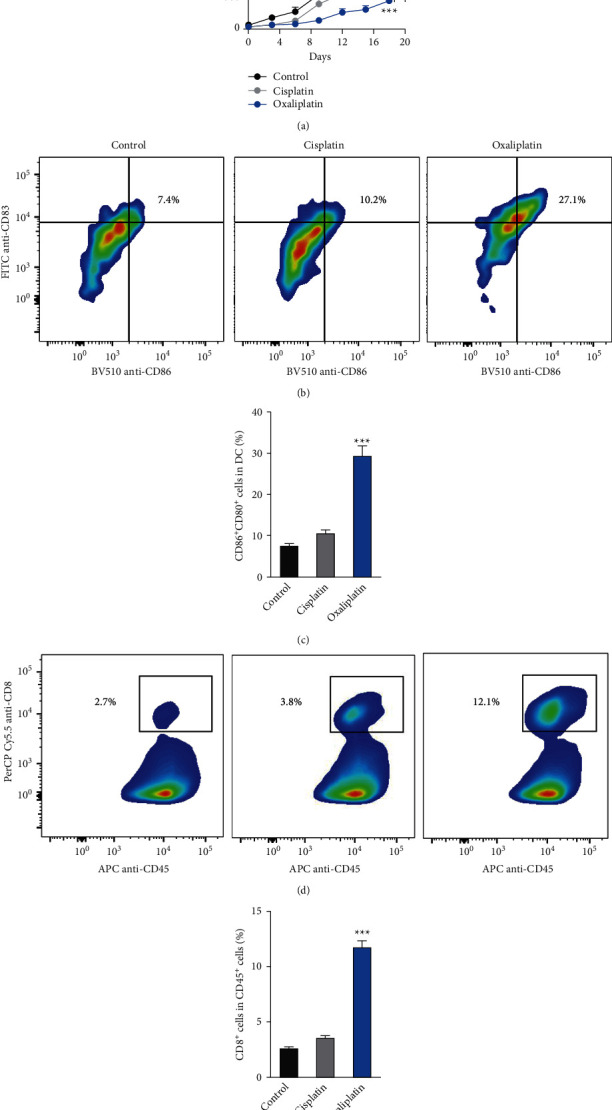
In vivo antitumor effect of cisplatin and oxaliplatin in a mouse model of head and neck tumor. (a) Inhibition of tumor growth by various treatments on C3H/HeJ mice bearing murine mouse head and neck carcinoma SSC7 (*n* = 6). When tumor volumes reached about 50 mm^3^, mice received PBS, cisplatin, or oxaliplatin every three days for 4 times, the administration dosage of cisplatin and oxaliplatin was 3.0 mg/kg (i.v.). Data were represented as the means ± SD. ^*∗*^*p* < 0.05 (versus cisplatin group). (b-c) Flow cytometer gating and histogram analysis of matured DCs in the tumor tissues at the end of treatment (*n* = 6). The matured DCs were denoted as CD80+ CD86+ populations (gate in CD45+ CD11b+ CD11c + cell population). (d-e) Flow cytometer gating and histogram analysis of cytotoxic T cells (CD8+ T cells) in the CD45+ tumor-infiltrating immune cells in tumor tissues from mice receiving indicated treatment (*n* = 6). ^*∗∗∗*^*p* < 0.001 compared with the cisplatin group.

Finally, we evaluated the effects of oxaliplatin on tumor growth in a xenograft animal model. The results showed that treatment with oxaliplatin (3.0 mg/kg, i.v.) significantly reduced the tumor volume as compared to the control and cisplatin-treated groups (Figure 5(a)). Next, we evaluated the populations of CD86^+^CD80^+^ and CD8^+^CD45^+^ cells in the tumor tissues. Interestingly, we observed that treatment with oxaliplatin (3.0 mg/kg, i.v.) significantly increased the populations of CD86^+^CD80^+^ (Figures 5(b) and 5(c)) and CD8^+^CD45^+^ cells (Figures 5(d) and 5(e)) in the tumor tissues as compared to the control and cisplatin-treated groups.

## 7. Discussion

This study investigated the antitumor activities of oxaliplatin against laryngeal cancer and its underlying mechanism. Our results showed that oxaliplatin inhibited cell growth and promoted cell apoptosis. In addition, oxaliplatin enhanced the phagocytosis effects of DCs against tumor cells and regulated the populations of tumor-specific T cells (IFN-*γ*-producing CD8^+^ T cells) and Treg cells. Taken together, these results supported that, the antitumor activities of oxaliplatin against laryngeal cancer were in part mediated by regulating ICD.

Cisplatin is one of the most commonly used chemo drugs in advanced laryngeal cancer therapy [21]. Antitumor activities of cisplatin are in part mediated by inducing DNA damage and promoting cell apoptosis [22]. However, cisplatin resistance is the major challenge in laryngeal cancer treatment [23]. Patients who initially respond to cisplatin therapy often develop acquired drug resistance after long-term exposure to cisplatin [23]. Oxaliplatin is a novel platinum derivative and belongs to the platinum class of drugs as cisplatin [24]. Oxaliplatin is featured by 1,2-diaminocyclohexane instead of two monodentate ammine ligands, which is different from cisplatin [24]. Enhanced replicated bypass and loss of mismatch repair are observed in cisplatin resistance. Enhanced replicated bypass has also been demonstrated for the cisplatin-DNA adduct, but not for the oxaliplatin-DNA adduct. In addition, the mismatch repair complex can recognize the cisplatin-DNA adduct, but not the oxaliplatin-DNA adduct [25]. In addition to structural differences, oxaliplatin exhibits stronger antitumor effects against gastric cancer than cisplatin in previous studies [26, 27]. To our knowledge, the antitumor activities of oxaliplatin against laryngeal cancer are still unknown. Therefore, this study was designed to investigate the antitumor activities of oxaliplatin against laryngeal cancer. Our results demonstrated that treatment with oxaliplatin reduced cell viability and promoted apoptotic cell populations in Hep-2 cells.

Interestingly, our results demonstrated that oxaliplatin showed similar antitumor efficacy as cisplatin. We further explored the underlying mechanisms of oxaliplatin and cisplatin against laryngeal cancer. Previous studies have demonstrated that cisplatin induces similar immunogenic changes as oxaliplatin in head and neck squamous cell carcinoma [28]. This study revealed that both cisplatin and oxaliplatin were weaker inducers of ICD in a xenograft mouse model. To our knowledge, the inducible effects of oxaliplatin on ICD have been reported in previous studies. For instance, Tesniere and colleagues found oxaliplatin-induced ICD in patients with colon cancer [29]. More recently, Zhu and colleagues reported that treatment with oxaliplatin-induced ICD in hepatoma cell lines showed synergistic effects with immune checkpoint blockage [16]. However, it is still unknown whether the antitumor activities of oxaliplatin and cisplatin against laryngeal cancer were associated with ICD. Herein, we investigated the effects of oxaliplatin and cisplatin on ICD. The results demonstrated that oxaliplatin, but not cisplatin, induced ICD in Hep-2 cells. These results supported that the underlying mechanisms of oxaliplatin against laryngeal cancer were different from cisplatin.

ICD is a complex process involved in many types of immune cells including macrophages, DCs, and T cells [18, 30, 31]. When ICD occurs, the activation of immune responses is accompanied by a series of cellular events [30]. The release of ATP serves as the “find me” signal for DCs [18]. In addition, ICD leads to the CRT translocation into the surface of cancer cells, which releases the “eat me” signal for DCs [18]. Furthermore, a large amount of HMGB1 is released during ICD which binds to the toll-like receptors on the immune cells (DCs and macrophages), leading to the release of inflammatory cytokines including IL-6 and TNF-*α* [18, 32]. Besides, HMGB1 is beneficial for enhancing the antigen-presenting functions of DCs [32]. Consequently, tumor-specific T cells are induced and T cell-mediated immune responses are triggered [33].

In the present study, we found that oxaliplatin, but not cisplatin, regulated a series of cellular events during ICD. First, treatment with oxaliplatin enhanced the phagocytosis of DCs against tumor cells and the maturation of DCs. Oxaliplatin enhanced the intensity of DiD in DCs and the levels of CD83 and CD86 on the surface of DCs. Second, treatment with oxaliplatin induced an inflammatory response in the coculture system. We observed that oxaliplatin increased the amount of ATP and HMGB1 and the levels of inflammatory cytokines including IL-6 and TNF-*α*. Third, treatment with oxaliplatin also regulated the population of T cells including tumor-specific T cells and Treg cells. Oxaliplatin increased the population of IFN-*γ*-producing CD8^+^ T cells and suppressed the population of CD4^+^CD25^+^FoxP3^+^ Tregs in the coculture system. However, several limitations must be noted. First, it would be of more clinical relevance if the findings are verified in patients. Second, detailed mechanisms could be explored to explain the underlying molecular events.

## 8. Conclusion

Oxaliplatin inhibited Hep-2 cell growth and regulated cell apoptosis. In addition, oxaliplatin-induced ICD in human laryngeal cancer cells by inducing the phagocytosis of DCs and regulating the populations of tumor-specific T cells and Treg cells. Taken together, oxaliplatin exhibits antitumor effects against laryngeal cancer in part by regulating ICD.

## Data Availability

All data generated or analyzed during this study are included in this article. The datasets used and/or analyzed during the current study are available from the corresponding author upon reasonable request.

## Supplementary Materials

Supplementary Materials Figure S1. Oxaliplatin induced immunogenic cell death (ICD) in AMC-HN-8 cells. (a) After the cells were incubated with cisplatin (7.5 *μ*M) or oxaliplatin (7.5 *μ*M) for 24 hours, AMC-HN-8 cells were stained with APC-labeled anti-CALR antibody. The levels of surface calreticulin (CALR) in viable cells (refers to as PI negative) were determined by a flow cytometer. (b) AMC-HN-8 cells were incubated with cisplatin (7.5 *μ*M) or oxaliplatin (7.5 *μ*M) for 24 hours, and then the levels of HMGB1 in cell supernatant were determined by Western blotting. BSA in the culture medium was used as the control protein. (c) The release of ATP was determined by using a commercialized kit. Data were represented as the means ± SD. ^*∗∗*^*p* < 0.01, ^*∗∗∗*^*p* < 0.001 as compared with the cisplatin group.
