# Supplementary material for: Oxaliplatin Induces Immunogenic Cell Death in Human and Murine Laryngeal Cancer
Source: J Oncol. 2022 Sep 12;2022:3760766. doi: 10.1155/2022/3760766 (PMC9484908; doi:10.1155/2022/3760766)
Supplement: Supplementary Materials — Figure S1. Oxaliplatin induced immunogenic cell death (ICD) in AMC-HN-8 cells. (a) After the cells were incubated with cisplatin (7.5 μM) or oxaliplatin (7.5 μM) for 24 hours, AMC-HN-8 cells were stained with APC-labeled anti-CALR antibody. The levels of surface calreticulin (CALR) in viable cells (refers to as PI negative) were determined by a flow cytometer. (b) AMC-HN-8 cells were incubated with cisplatin (7.5 μM) or oxaliplatin (7.5 μM) for 24 hours, and then the levels of HMGB1 in cell supernatant were determined by Western blotting. BSA in the culture medium was used as the control protein. (c) The release of ATP was determined by using a commercialized kit. Data were represented as the means ± SD. ∗∗p < 0.01, ∗∗∗p < 0.001 as compared with the cisplatin group. [file 3760766.f1.docx]

**Supplementary materials**

**Figure S1. Oxaliplatin induced immunogenic cell death (ICD) in AMC-HN-8 cells.** (A) After the cells were incubated with Cisplatin (7.5 μM) or Oxaliplatin (7.5 μM) for 24 hours, AMC-HN-8 cells were stained with APC-labeled anti-CALR antibody. The levels of surface calreticulin (CALR) in viable cells (refers to PI negative) were determined by a flow cytometer. (B) AMC-HN-8 cells were incubated with Cisplatin (7.5 μM) or Oxaliplatin (7.5 μM) for 24 hours, and then the levels of HMGB1 in cell supernatant were determined by western blotting. BSA in the culture medium was used as the control protein. (C) The release of ATP was determined by using a commercialized kit. Data were represented as the means ± SD. ***p* < 0.01, ****p* < 0.001 as compared to Cisplatin group.
